# Anti-Idiotypic Antibodies in Immune Regulation and Disease: Therapeutic Promise for Next-Generation Vaccines

**DOI:** 10.3390/vaccines13121224

**Published:** 2025-12-03

**Authors:** Anna M. Timofeeva, Sergey E. Sedykh, Georgy A. Nevinsky

**Affiliations:** SB RAS Institute of Chemical Biology and Fundamental Medicine, 630090 Novosibirsk, Russia

**Keywords:** anti-idiotype, idiotype, anti-idiotypic antibodies, antibodies, vaccines, cancer vaccination, oncology, HIV-1, catalytic antibodies, Jerne network, anti-drug antibodies

## Abstract

**Background:** Antibodies have the unique ability to recognize antigens and to be recognized as antigens by other antibodies, creating a balanced network that regulates the humoral part of the immune system. An antibody that uniquely identifies another antibody of a given specificity as its antigen is referred to as an anti-idiotypic antibody. **Methods:** A descriptive literature review was conducted using the PubMed database, including publications up to 2025. **Results:** This review examines the formation mechanisms of anti-idiotypic antibodies, their functional attributes, and their importance in diverse pathologies. A key focus is their capacity to neutralize pathogenic autoantibodies, offering a novel strategy for treating autoimmune diseases. Conversely, the generation of anti-Id Abs against therapeutic monoclonal antibodies (anti-drug antibodies) represents a significant challenge for biologic therapy, a complication addressed in a dedicated section on detection methods. Furthermore, consideration is given to the application of anti-Id Abs as innovative tools for vaccine design, particularly in oncology. By mimicking tumor-associated antigens, anti-Id Abs can induce a potent, targeted immune response against cancer with minimal side effects, presenting an alternative to conventional chemotherapy and radiation. **Conclusions:** Anti-Id Abs hold significant therapeutic promise. Their ability to selectively suppress pathogenic autoantibodies allows for precise immune intervention without broad immunosuppression. Additionally, their utility extends to vaccine development for various diseases. Further research into anti-Id Abs will deepen our understanding of immune regulation and open new avenues for targeted therapies.

## 1. Introduction

**Figure 1:**
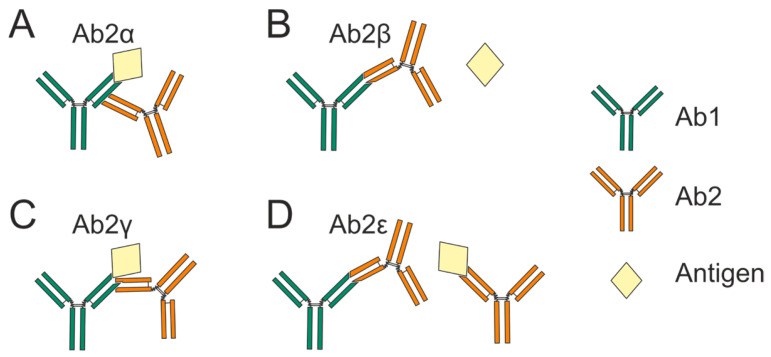
Classification of anti-Id Abs according to the location of the paratope and idiotope on Ab1 and Ab2 antibodies. Anti-Id Abs can be categorized into four classes: Ab2α (**A**), Ab2β (**B**), Ab2γ (**C**), and Ab2ε (**D**).

The immune system is regulated by a complex network of interactions, one of the most intriguing being the ability of antibodies to recognize other antibodies. An antibody that uniquely identifies another antibody of a given specificity as its antigen is referred to as an anti-idiotypic antibody (anti-Id Ab). Experimental and clinical studies have shown that animals and humans are capable of producing anti-Id Abs to their own immunoglobulins [1]. The anti-idiotypic antibody (Ab2) can target different epitopes of the parent antibody (Ab1). The established classification in terms of the binding sites of anti-Id Abs comprises four main classes: Ab2α, Ab2β, Ab2γ, and Ab2ε (Figure 1):

(a) Ab2α binds outside the Ab1 antigen-binding site.
(b) Ab2β antibodies bind specifically to the complementarity-determining region (CDR) of Ab1.This allows them to functionally mimic the antigen and directly compete with it for occupancy of the Ab1 binding site.
(c) Ab2γ binds to the paratope or near the paratope of Ab1, where it sterically occludes antigen access and blocks binding [2,3].
(d) Ab2ε antibodies demonstrate dual reactivity by recognizing both Ab1 and the cognate antigen itself [4]. Subsequent research has revealed antibodies with expanded polyreactivity that, while similar to Ab2ε, also possess self-binding activity in addition to anti-idiotypic and antigen-binding capabilities [5,6].

Among these classes, Ab2β antibodies are of paramount interest and are a primary focus of this review. These anti-Id Abs recognize the binding site of the parent antibody and thus prevent the parent antibody from binding to its antigen [7]. Also, Ab2βs possess an “internal image” of the external antigen, which leads to anti-Id Abs and antigens competitively binding to specific antibodies [8].

This review aims to synthesize the current understanding of anti-Id Abs. We begin by examining their fundamental characteristics and generation mechanisms (Section 2), followed by their roles in various pathological conditions (Section 3). We then explore their dual significance in therapy: on one hand, their ability to neutralize pathogenic autoantibodies presents a promising avenue for treating autoimmune diseases; on the other hand, their formation as anti-drug antibodies can compromise the efficacy of monoclonal antibody therapies (Section 4). Another remarkable aspect under discussion is the ability of certain anti-Id Abs to mimic biologically active molecules, such as cytokines, neurotransmitters, and hormones, acting as their functional internal images (Section 5). Finally, we highlight the most translational application of these antibodies: their potential as innovative tools for vaccine development, particularly in the fields of oncology and infectious diseases like HIV (Section 6). By consolidating this knowledge, this review underscores the broad prospects of anti-Id Ab research for developing novel targeted therapies.

## 2. Network Concept and Idiotypic Cascades

In 1974, Niels Kaj Jerne proposed the “network hypothesis” [8]. He postulated that antibodies have the unique ability to both recognize antigens and to be recognized by other antibodies as antigens, thus creating a balanced network that regulates the humoral part of the immune system [9]. Immunological homeostasis involves an equilibrium between the primary antibody (idiotypic antibody, Ab1) and the secondary antibody (anti-idiotypic, anti-Id, Ab2), with Ab2-reactive lymphocytes governing the formation of Ab1. Anti-Id Abs are responsible for maintaining the homeostasis of adaptive humoral immune responses via the neutralization of idiotypic antibodies and the regulation of their secretion [10,11]. An Anti-Id Ab, possessing an idiotype similar to the epitope of the antigen, can bind to the antigen-binding site of Ab1 and compete with the antigen for binding to Ab1, thereby regulating the function of the antibody.

The network concept was investigated in several separate studies, with Ab2β used as an antigen to generate specific immune responses [12,13]. Research has shown that interactions between idiotypic antibodies and anti-Id Abs can regulate the immune response to an antigen, either stimulating or inhibiting it, according to research [14,15]. When the body encounters its own or foreign antigens, the immunologic equilibrium is altered, which causes Ab1 to be generated. Therefore, the generation of Ab2 antibodies is increased. Ab2 regulates the function and variety of Ab1-producing B cells, resulting in lower levels of Ab1 [16]. In other words, the immune response can be modulated, either dampened or heightened, by the generation of anti-Id Abs. For example, tetanus anatoxin vaccination has been shown not only to induce the expected increase in antibodies against tetanus anatoxin but also to trigger a subsequent increase in anti-Id Abs that reduce to normalize the level of free antibodies to tetanus anatoxin [17].

**Figure 2:**
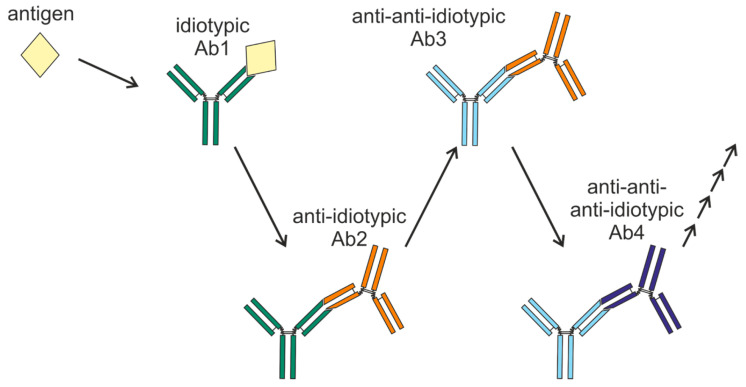
Formation mechanism of the anti-idiotypic antibody cascade. The antigen elicits the formation of idiotypic Ab1 antibodies. These antibodies can potentially trigger the generation of anti-idiotypic Ab2 antibodies, which can then induce the formation of anti-anti-idiotypic Ab3 antibodies, and so on.

Anti-idiotypic responses can be induced not only by Ab1 idiotypic antibodies but also by anti-Id Abs (Ab2) (Figure 2). To illustrate, anti-anti-Id Abs (Ab3) were generated in rabbits immunized with polyclonal Ab2 induced by purified Ab1 produced in response to carbohydrate immunization of *Micrococcus lysodeikticus* bacteria [18]. Comparable results were observed in BALB/c mice that were immunized with myeloma levan-binding protein. These mice produced not only Ab2 antibodies but also Ab3 antibodies, which were further used for the induction of anti-anti-anti-Id Abs (Ab4) [19]. The Ab1 and Ab3 members of this idiotypic cascade are linked by a shared cross-reactive regulatory idiotope that interacts with both Ab2 and Ab4 [19]. The same sequential progression of the idiotypic cascade has also been identified in humans, specifically in cancer patients undergoing treatment with therapeutic antibodies [20]. Idiotypic cascades have been reported to result in anti-anti-anti-anti-idiotype (Ab4) and even in Ab6 [21]. Figure 2 presents a schematic representation of the anti-Id Ab cascade formation.

The study of idiotypic cascades has yielded an important discovery: although antigen-induced Ab1 can provoke an anti-idiotypic response (Ab2), Ab2 immunization does not result in Ab1 recruitment. The subsequent stages of the cascade also follow this rule. Hence, the idiotypic cascade moves only forward. The Ab3 response to Ab2 is different from that to Ab1.

### Similarity in Epitopes Between Antigens and Anti-Idiotypic Antibodies

The interaction between Ab1 and anti-Id Ab is a perfect fit, similar to a key and its lock. These interactions are studied by examining the 3D structure. Multiple research efforts have revealed the 3D structures of different anti-Id mAbs, both independently and when bound to an idiotope [22,23,24,25]. Hen egg lysozyme (HEL) serves as the antigen for monoclonal antibody D1.3. The three-dimensional crystal structures of antibody D1.3 complexes with HEL [26] and anti-Id mAb (E5.2) [27] have been determined. The three-dimensional structures of the two complexes were compared, revealing that 18 amino acid residues of antibody D1.3 participated in binding to anti-Id mAb E5.2, whereas 17 residues were involved in antigen binding. Furthermore, 13 amino acid residues were found to contribute to the formation of bonds with both anti-Id mAb and antigen. These 13 amino acid residues of D1.3 were identified to account for 75% of the antibody contact area with the anti-Id mAb and 87% of the antigen contact area, with the contact patterns comparable in each case. Hence, anti-Id mAb E5.2 has been observed to imitate the HEL antigen, despite not presenting a precise “topological image” [27]. These findings were acquired exclusively through the utilization of a structural approach.

Anti-Id Ab establishes chemical contacts with Ab1 residues that are essentially identical to those that interact with the antigen. Nonetheless, this does not indicate a structural similarity between anti-Id Ab and the antigen. The mimicry of anti-Id Ab epitopes is functional and mediated by similar binding interactions [27]. This nonstructural but functional mimicry is also observed with anti-Id Abs responding to Ab1 generated against non-protein antigens. For example, Ab2 is unable to mimic polysaccharide structure, but it can trigger a polysaccharide-specific reaction to a polysaccharide antigen [28].

## 3. Anti-Idiotypic Antibodies in Normal and Pathological Conditions

### 3.1. Autoimmune Diseases: The Balance Between Autoantibodies and Anti-Idiotypic Antibodies

Autoimmune diseases arise from a breakdown in immune tolerance, leading the immune system to mistakenly target and damage the body’s own tissues. For example, systemic lupus erythematosus (SLE) is a chronic autoimmune disease, which is defined by the production of autoantibodies against a wide range of self-antigens, including nuclear antigens (nucleosomes, DNA, histones), myelin sheath proteins, phospholipids, and others [29].

The potential for anti-idiotypic antibodies to bind and regulate autoantibody levels could have beneficial effects on disease progression. This is corroborated by research demonstrating the absence of anti-Id Abs during periods of active autoimmune disease [30]. Anti-Id Abs against autoantibodies have been described in several autoimmune diseases such as SLE, myasthenia gravis, Sjögren’s syndrome, and idiopathic thrombocytopenic purpura [31,32,33,34].

Autoantibodies against double-stranded DNA (dsDNA) are a serological hallmark of most commonly detected in SLE. High levels of anti-Id Abs to anti-dsDNA antibodies are found during remission in patients with autoimmune diseases, with lower levels of anti-Id Abs antibodies found during the acute, active phases of the disease [35,36]. It is worth noting that the concentrations of anti-Id Abs frequently exhibit an inverse relationship with the activity of autoimmune diseases [7]. Notably, anti-Id Abs against anti-dsDNA antibodies are found in the family members of those with SLE [37], people who interacted with SLE patients [38], and even in healthy individuals [39].

It has also been demonstrated that autoimmune sera from patients with primary Sjögren’s syndrome and SLE contain anti-Id Abs, anti-La/SSB antibodies, which inhibit the binding of anti-La/SSB antibodies to recombinant La/SSB by 91% [34].

Anti-Id Abs have also been described in the normal human immune response. It is suggested that they provide protection by obstructing pathogenic autoantibodies [17]. In other words, autoantibodies are present in healthy individuals but are masked by the presence of anti-Id Abs. Consequently, despite the fact that most healthy individuals test negative for autoantibodies with standard antibody assays, more advanced assays reveal the presence of autoantibodies that are concealed by anti-Id Abs [40]. Let us proceed with particular examples. It is believed that anti-P autoantibodies are specific to systemic lupus erythematosus patients. However, anti-P antibodies were readily identified in healthy participants who were serologically negative for anti-P antibodies during routine screening, once inhibitory antibodies had been removed [41,42].

Another case is that autoantibodies against the E2 subunit of the pyruvate dehydrogenase complex are considered characteristic of patients suffering from primary biliary cirrhosis. However, they are also present in healthy individuals but are masked by anti-Id Abs [43].

One more illustration is that antibodies to the 65-kDa isoform of glutamate decarboxylase (GAD65) are markers of autoimmune response in type 1 diabetes (DM1) and latent autoimmune diabetes in adults (LADA) [44,45]. In healthy individuals, GAD65 antibodies are found in a complex with GAD65-specific anti-Id Abs. Consequently, these antibodies are undetectable in standard assays. Using immobilized monoclonal antibodies against GAD65 to absorb anti-Id Abs from plasma enabled the detection of GAD65 antibodies in serum [44]. Within the autoimmune process, the extended release of GAD65 during beta cell destruction leads to the permanent activation of polyclonal GAD65-specific B cells and subsequent secretion of anti-GAD65 antibodies. Alterations in secreted anti-GAD65 antibody idiotypes will result from somatic mutations and affinity maturation, ultimately leading to an inadequate anti-idiotypic response to these modifications. The concentration of anti-Id Abs specific to GAD65 autoantibodies was reported to be significantly lower in DM1 patients relative to healthy individuals [44]. Upon remission, patients with DM1 exhibit elevated levels of anti-Id Abs [46]. Furthermore, a reduction in anti-GAD65 autoantibodies has been observed in the early stages of DM1 development, preceding the emergence of additional markers associated with the autoimmune response [7]. Hence, anti-Id Abs to GAD65 autoantibodies can be used to predict DM1.

Consequently, the equilibrium of autoantibodies and anti-Id Abs supports the homeostasis of the adaptive immune response. The disruption of this equilibrium, caused by reduced protective elements or elevated autoimmunity, may cause autoimmune diseases.

A noteworthy study concerning the elicitation of autoimmunity in immunologically naïve mice through idiotypic immunization has been reported. (1) Following administration of Id-16/6 anti-dsDNA antibodies, mice exhibited SLE symptoms; (2) immunization with anti-cardiolipin antibodies (H-3 and MIV-7) led to the development of antiphospholipid syndrome; and (3) Wegener’s granulomatosis was induced by anti-neutrophil cytoplasmic antibodies. Across all three experiments, mice generated corresponding anti-Id Abs (Ab2), and subsequently, within 4–8 months, immunized mice produced anti-anti-Id Abs (Ab3). The emergence of these antibodies in the serum of the mice correlated with the emergence of all classical clinical and laboratory indicators observed in individuals with these autoimmune disorders [47].

### 3.2. Therapeutic Applications of Anti-Idiotypic Antibodies in Autoimmune Diseases

Treatment strategies for autoimmune diseases typically involve either nonspecific immunosuppression or the targeting of disease manifestations [48]. However, autoimmune disease management involves extended therapy with elevated dosages of immunosuppressants, which can cause life-threatening opportunistic infections and undesirable adverse effects [49]. Therefore, the past few years have witnessed attempts to employ anti-Id Abs for the treatment of autoimmune diseases.

Antibodies against IgG constant regions, also known as rheumatoid factor [50,51], are used as one of the markers for the early diagnosis of rheumatoid arthritis. Anti-Id Abs against monoclonal rheumatoid factor have been developed and were reported to significantly decrease rheumatoid factor production in vitro in cell cultures from rheumatoid arthritis patients [52].

Administration of human monoclonal antibody b96.11, specific to GAD65, to prediabetic and non-obese diabetic (NOD) mice substantially delays the onset of autoimmune diabetes. This treatment also resulted in the formation of anti-Id Abs. These induced anti-Id Abs were found to block GAD65 binding to the corresponding antibody [53]. This finding is consistent with observations in humans, where the presence of both GAD65-specific anti-Id Abs and GAD65 autoantibodies has been observed in the serum of healthy subjects [44].

### 3.3. Formation of Catalytic Antibodies via an Anti-Idiotypic Mechanism

**Figure 3:**
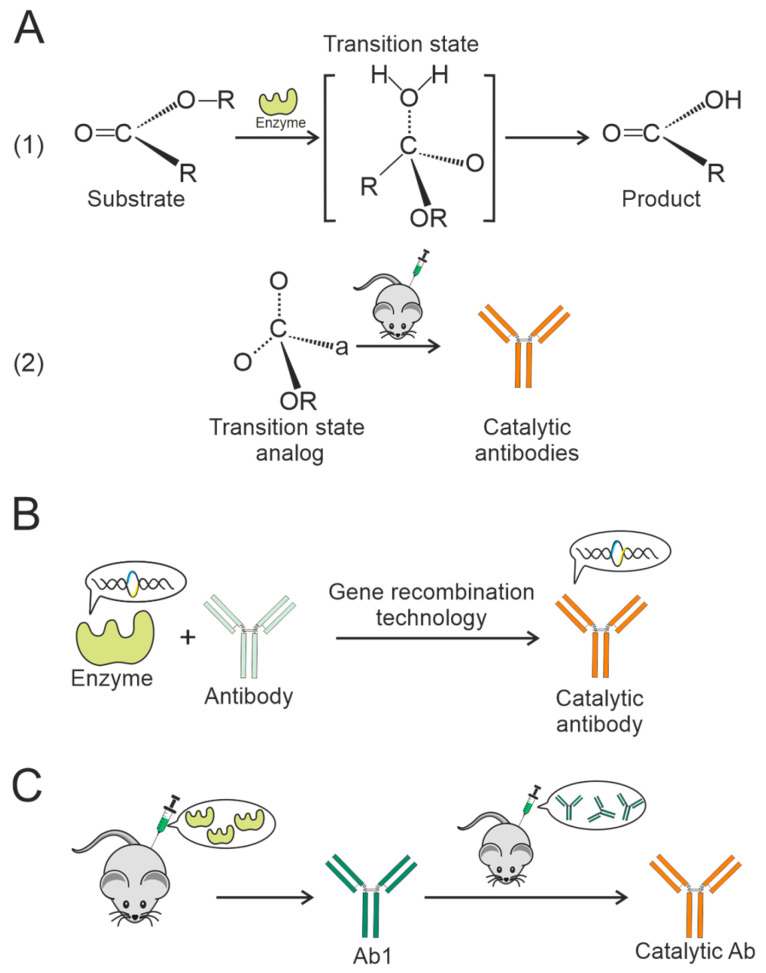
Various methods for producing catalytic antibodies: (**A**) Synthesis of stable transition state analogs (**1**) and their use as haptens for animal immunization (**2**) [82,83]. (**B**) Production of antibodies with catalytic function by recombining or replacing a fragment of the catalytic domain of an enzyme with a variegated region of the antibody [82]. (**C**) Generation of catalytic antibody based on “idiotypic network theory” [82,84].

Catalytic antibodies, which can not only bind antigens but also catalyze specific reactions in a manner similar to enzymes, have been known to exist for a considerable amount of time [54,55,56,57]. Lerner postulated in 1984 [58] that antibody-derived transition state analogs are capable of inducing catalysis by forcing the substrate into the transition state following binding (Figure 3A). This hypothesis suggests that antibodies developed against a tetrahedrally charged phosphate hapten can catalyze the hydrolysis of corresponding carbonate and carboxylic acid esters [59,60]. Subsequently, natural antibodies with the ability to cleave nucleic acids, proteins, and polysaccharide substrates were documented. The presence of such catalytic antibodies that bind to host antigens has often been associated with various autoimmune diseases. For example, patients with autoimmune conditions such as systemic lupus erythematosus, scleroderma, rheumatoid arthritis, and multiple sclerosis were found to possess catalytic antibodies that hydrolyze DNA, RNA, and some specific autoantigens [29,61,62,63]. Proteolytic antibodies, both polyclonal [64,65] and monoclonal [66,67,68], targeting β-amyloid in Alzheimer’s disease [69], have been discovered. Furthermore, IgG and IgM have been shown to hydrolyze HIV proteins such as gp120 [70,71,72,73], gp41 [74], and other substrates [75,76].

Further research has led to the development of various methods and techniques for generating catalytic antibodies with specific characteristics, especially those not found in nature [77,78]. One approach to generating catalytic antibodies involves creating haptens that replicate transition-state characteristics in catalytic reactions. Among the disadvantages of this approach are weak antibody activity and inefficient mimicking of natural enzymes [79,80,81].

An alternative strategy is the production of catalytic anti-idiotypic antibodies (anti-Id Abs). In this approach, a natural enzyme is used as the antigen [85,86] (Figure 3B). According to Jerne’s network concept [8], the active site of an enzyme can be mimicked through sequential immune responses. The first antibody (idiotypic, Ab1) is generated against the enzyme’s active site. The second antibody (anti-idiotypic, Ab2), which is complementary to Ab1, then mirrors the structure and function of the original enzymatic site (Figure 3C). This method has been successfully used to generate antibodies with amidase [87], serine protease [88], esterase [89], and carboxypeptidase activity [90].

Specific examples illustrate this principle. The anti-idiotypic monoclonal IgG antibody 9G4H9 was generated against an antibody that recognizes the active site of β-lactamase. The resulting 9G4H9 antibody was shown to exhibit β-lactamase-like activity, despite having no sequence homology with natural β-lactamases [91]. Similarly, the anti-idiotypic monoclonal IgM antibody 9A8, produced against an acetylcholinesterase-inhibiting mAb (AE-2), was found to possess esterolytic activity. Again, its variable domain demonstrated catalytic function without sharing primary sequence homology with the enzyme [92].

The discovery of nanobodies—single-domain antibodies composed solely of heavy chains found in *Camelidae* [93]—has provided a powerful platform for generating catalytic anti-Id Abs. Their simple architecture facilitates the construction of robust single-domain antibody libraries [94]. Such phage display libraries can be screened using Ab1 as an antigen to select for catalytic anti-Id Abs (Ab2), a class of antibodies that has been shown to be well-represented in these libraries [78]. For instance, a catalytic anti-Id nanobody was obtained that converts the substrate alliin into the biologically active product allicin [78].

### 3.4. Anti-Idiotypic Antibodies in COVID-19

The receptor-binding domain (RBD) of the virus is located in the spike protein and plays an important role in viral binding to the host cell receptor [95]. SARS-CoV-2 uses the catalytic site of the ACE2 receptor for viral entry through the process of endocytosis [95].

It has been reported that following SARS-CoV-2 infection, not only are antibodies against the SARS-CoV-2 S-protein produced, but also autoantibodies against ACE2, which may be significant for the development of long COVID after SARS-CoV-2 infection [96,97,98,99,100]. Antibodies against ACE2 are detected in the blood of individuals who have recovered from COVID-19, and their titer significantly correlates with the severity of the disease [101]. Moreover, the presence of anti-ACE2 antibodies is not detected prior to SARS-CoV-2 infection [102].

Mouse studies have shown that serial vaccination with mRNA-1273 against SARS-CoV-2 induces not only persistent anti-spike antibodies but also antibodies against ACE2 in both wild-type mice and K18 mice (transgenic mice expressing human ACE2). Importantly, the anti-idiotypic response is polyclonal in nature, and anti-ACE2 antibodies constitute only a subset of the overall anti-Id Abs population [103].

Viewed through the lens of Jerne’s anti-idiotypic network hypothesis [104], anti-ACE2 antibodies are anti-idiotypic and may be structurally similar to the original antigen (the RBD of the SARS-CoV-2 Spike protein) and, consequently, capable of binding to the same receptors as the original antigen [98].

It is worth noting that anti-ACE2 antibodies can influence the results of S-protein antibody titer assays. This occurs due to competition between the S-protein coated in the plate wells and the anti-ACE2 antibodies for binding to anti-spike antibodies. Thus, these anti-ACE2 antibodies can lead to an underestimation of the quantitative determination of anti-S antibodies in a sample [105]. Furthermore, it has been shown that some COVID-19 patients produce antibodies with catalytic activity capable of cleaving a synthetic ACE2 substrate [106] and the RBD [56].

## 4. Antibodies with Anti-Drug Activity

Modern technology has facilitated the active development of monoclonal antibodies (mAb) as pharmaceutical agents [107,108]. Monoclonal therapeutic antibodies are a versatile class of therapeutics for treating a wide range of human diseases due to their high specificity. To date, monoclonal antibodies are one of the most promising agents in the pharmaceutical industry, constituting nearly half of the therapeutic proteins approved by the U.S. Food and Drug Administration (FDA) and the European Medicines Agency (EMA) [109]. Hundreds more mAbs are in various stages of clinical development.

Despite the clinical and commercial success of numerous antibody-based treatments, their effectiveness tends to be restricted to a subset of patients. A major challenge for therapeutic antibodies is their immunogenicity. The immunogenicity of therapeutic mAbs primarily manifests in the formation of anti-Id Abs. Such antibodies are called anti-drug antibodies (ADAs) [110]. The development of ADAs alters the bioavailability, pharmacokinetic, and pharmacodynamic properties of therapeutic mAbs and reduces their efficacy [111,112]. Numerous research investigations have demonstrated an inverse correlation between the production of ADAs as a result of treatment, the functional levels of the particular medication administered, and the clinical response observed, such as in the cases of natalizumab [113] (anti-α4-integrin), infliximab [114,115], and adalimumab [116,117]. Due to the possibility of causing severe adverse immune reactions, ADAs can have a negative impact on the safety profile of mAb drugs [118]. The antigenic site of ADA is most often associated with the binding region of the antibody. However, the generation of ADAs targeting the hinge regions of the antibody has also been reported following treatment with abatacept and etanercept [119].

Adalimumab, infliximab, and golimumab, which are anti-TNFα drugs for immune-mediated diseases, are the best examples to characterize the development of ADA [120]. The prevalence of ADA formation differs depending on the specific drug, with infliximab exhibiting a greater incidence of ADA development (0–83% of patients) relative to adalimumab (0–54%) and golimumab (0–19%) [119].

Immune checkpoint inhibitor mAbs targeting PD-1 (pembrolizumab and nivolumab) are approved for use in metastatic melanoma, renal cell carcinoma, and small cell lung cancer. ADAs against pembrolizumab have been reported to occur at a frequency of 0.7–2.5% [121,122,123].

Infliximab is a drug approved for the treatment of rheumatoid arthritis. However, the emergence of ADAs with neutralizing activity is a concern with infliximab use: the reported prevalence of ADAs in patients undergoing therapy ranges from 15 to 54% [114,124,125]. ADAs to rituximab have been reported in 4–11% of patients treated for rheumatoid arthritis [114] and in 26–37% of patients treated for multiple sclerosis [113].

There is another challenge that researchers face in developing therapeutic mAbs against HIV. Monoclonal antibody efficacy studies are often conducted on macaques infected with SHIV [126,127,128]. A limitation of these types of studies is the propensity for ADA formation when human mAbs are introduced to rhesus macaques, given that the whole antibody (Fab and Fc domains included) is recognized as foreign [129].

In summary, it is important to emphasize that the immunogenicity of therapeutic mAbs can be influenced by many factors, including product-specific factors (e.g., protein structure), treatment-related factors (e.g., use of concomitant therapy, dosing, continuous or intermittent administration), and patient-related factors (e.g., genetic predisposition and underlying disease). For example, the rate of ADA formation has been shown to be higher with chimeric mAbs compared to some fully human mAbs and fusion proteins [119]. Currently, many gaps remain in the understanding of ADAs.

### Techniques for Detecting Anti-Drug Antibodies

Enzyme-linked immunosorbent assays (ELISAs) are widely employed for ADA screening, including direct assays [130], sandwich assays [131], bridging assays [132], and competitive ELISAs [133]. However, the detection of ADA via this method is complicated by the presence of drugs and ADA–drug complexes in the blood.

ELISA can detect anti-idiotypic antibodies using sorbed idiotypic antibodies in plate wells. Given that anti-Id Abs are usually in equilibrium with idiotypic antibodies, the detection of such antibodies in plasma demands a pretreatment step designed to deplete the target idiotypic antibodies [134]. An affinity-based technique employing sorbed antigen can be used to purify plasma from masking idiotypic antibodies, as demonstrated in [29,36].

Alternative approaches for the detection of ADA encompass high-performance liquid chromatography [135], electrochemiluminescent assays [136,137], radiometric assays [138], radioimmunoassays [139], and cytotoxicity assays [140].

ADA–drug complexes can be isolated by acidifying plasma samples [141]. An alternative is antigen-binding testing, which measures the quantity of ADA–drug immune complexes [110]. For example, the feasibility of using liquid chromatography and tandem mass spectrometry (LC-MS/MS) to measure ADA–drug immunocomplexes has been demonstrated using infliximab as an example. This approach relies on the use of isotopically labeled antigen-binding fragments of infliximab (SIL_IFX_F(ab’)2) for indirect binding and ADA quantification. Following the capture of IgG, including ADA, with Protein A magnetic beads, SIL_IFX_F(ab’)2 was added for labeling purposes. Subsequently, the ADA concentration was measured using LC-MS/MS [142].

## 5. Anti-Idiotypic Antibodies That Can Mimic Diverse Molecules

Anti-Id Abs have been engineered to emulate a wide range of molecules, including interferons, neurotransmitters, hormones, and substances often associated with drug abuse. The following section will delve into various examples of such antibodies.

A monoclonal antibody known as D9D10 functions by inhibiting the biological activity of human interferon-γ, often referred to as IFN-γ. An anti-Id mAb AA1E5, which embodies the intrinsic image of IFN-γ, was generated. AA1E5 exhibits competitive binding with IFN-γ at D9D10, which is indicative of its anti-idiotypic nature. Moreover, the AA1E5 molecule mirrors human IFN-γ by binding to the IFN-γ receptor [143]. Sequence comparison between the complementarity-determining regions of the antibody and IFN-γ revealed that both the heavy chain variable domain (VH) and the kappa light chain variable domain contain 3–4 amino acid epitopes that are also present in the IFN-γ sequence and contribute to receptor binding [144].

Anti-Id mAbs against antibodies to haloperidol (neurotransmitter) were obtained. These anti-Id mAbs exhibited an effect comparable to haloperidol on dopamine D-2 receptors and exhibited successful binding [145].

Another study involved immunizing rabbits with affinity-purified antibodies against serotonin (5-hydroxytryptamine) to obtain anti-Id Abs. The resulting anti-Id Abs were found to bind to complete anti-5-HT antibodies, as well as their Fab fragments. The findings indicated that these anti-Id Abs recognized 5-HT1B, 5-HT1C, and 5-HT2 subtype receptors [146].

In a further example, anti-Id Abs were produced by using rat insulin-specific antibodies as an immunogen. These antibodies successfully blocked the interaction of insulin with its receptor. The binding of anti-Id antibodies to thymocyte insulin receptors functionally mirrored insulin binding, as measured by α-aminoisobutyric acid uptake [147].

Furthermore, the ability of anti-Id mAb 1D5 to interact with the estrogen receptor was assessed by examining its binding to the site of a monoclonal anti-estradiol antibody. The data revealed that 1D5 could replicate estradiol bioactivity through the recognition of estradiol receptors [148].

The development of anti-Id mAb B-32 specific for growth hormone was reported in another study. The function of B-32 involves specific interactions with growth hormone receptors, activated growth hormone receptors and Janus kinase signal transducers, and the JAK2/STAT5 signaling pathways [149].

Monoclonal antibodies to morphine were used to generate a mouse anti-idiotypic mAb (Ab2-AOR) targeting the opioid receptor. In competitive binding assays, Ab2-AOR was shown to compete with tritiated opioid ligands—dihydromorphine, naloxone, and etorphine—for rat brain opioid receptors [150]. In a separate study, anti-Id Abs were developed that functionally mimic the morphine molecule. The authors demonstrated that these antibodies inhibit the binding of morphine to both opiate receptors and anti-morphine antibodies [151].

An anti-Id mAb K2-3f was obtained that mimics the cocaine molecule and binds to hDAT with higher affinity than cocaine itself. Furthermore, K2-3f not only prevents cocaine binding to hDAT but concurrently promotes dopamine uptake [152].

## 6. Therapeutic Potential of Anti-Idiotypic Antibodies in Vaccine Development

The idiotype-based approach can serve as a foundation for vaccine development. This approach involves the use of anti-Id Abs as antigen-mimetics to induce a specific immune response against the target antigen. The anti-Id Abs mimic the shape of a key part of the antigen, resulting in the immune system producing antibodies against the target antigen. Anti-Id Abs have the potential for immunization against conformation-dependent epitopes and low-immunogenic, non-protein antigens. Anti-Id Abs can be designed to mimic lipid, carbohydrate, nucleic acid epitopes, or drugs [153]. In such cases, anti-Id Abs are proteins that mimic a non-protein epitope [154]. Furthermore, an anti-idiotypic vaccine may reduce the probability of undesirable adverse effects that are frequently linked to standard antigenic vaccines [155]. Given appropriate selection, monoclonal anti-Id Abs (anti-Id mAbs) can substitute for the antigen without eliciting a broad spectrum of antibodies in the same manner as the original antigen [156]. Another advantage of the anti-idiotypic vaccine is its high specificity, which allows for focusing the immune response on a critical neutralizing epitope.

The following section reviews some examples of anti-idiotype antibodies that could be used in vaccine development.

### 6.1. Antidiotypic Vaccines Targeting Tumor-Associated Carbohydrate Antigens

Abnormal glycosylation is connected to the expression of carbohydrate-based tumor-associated antigens on tumor cells [157]. Gangliosides play a significant role in cancer immunotherapy efforts. The National Cancer Institute researched and identified 75 potential antigens for cancer vaccines, with four of them being gangliosides GD2, GD3, fucosyl GM1, and N-acetyl-GM3 [158].

Although gangliosides have weak immunogenic properties, several studies have revealed the presence of endogenous anti-ganglioside antibodies. Antibodies targeting GM2 and GD2 were identified in the serum of melanoma patients and healthy individuals [159]. A separate study has reported the presence of anti-GM1, anti-GD1a, anti-GD1b, and anti-GT1b antibodies in healthy subjects [160]. In addition, anti-ganglioside antibodies have been shown to have antitumor cytotoxic ability [161]. Antibody binding to ganglioside causes conformational alterations, thereby triggering intracellular signals that promote apoptosis [162]. Anti-GD2 antibodies from healthy donors have also been reported to be cytotoxic against neuroblastoma cells [163].

Ganglioside-mimicking anti-Id Abs were developed for the purpose of tumor immunotherapy. In rabbits, immunization with mouse anti-Id Ab BEC2 versus anti-GD3 mAb R24 demonstrated GD3-mimicking ability and induced a specific antibody response [164]. In clinical trials involving melanoma and small cell lung cancer patients, BEC2 exhibited immunogenicity and stimulated an anti-anti-idiotypic response. However, only a minority of patients developed specific anti-GD3 antibodies [164] (Table 1).

Another study involved the production of an anti-Id Ab that mimics GD2 ganglioside. A specific IgG response against ganglioside was elicited upon immunization with this antibody, leading to the induction of lysis in GD2-positive cells [165]. Based on anti-Id mAbs, a therapeutic drug TriGem has been developed that mimics the disialoganglioside GD2, which is highly expressed in melanomas. The preliminary study indicated that of the 12 patients treated, one exhibited a complete response, while six demonstrated a cessation of tumor progression. The subsequent research revealed a complete response in one patient, and 12 out of 40 patients had stable disease [166]. TriGem has undergone evaluation in both preclinical and clinical settings [166].

Racotumomab, Ab2γ, was produced against the murine anti-ganglioside N-glycolyl (NGc) GM3 (NGcGM3) [167]. The safety profile of racotumomab has been demonstrated in multiple Phase I studies across melanoma, breast, and lung cancers [168,169]. Patients who generated antibodies against NGcGM3 were found to exhibit extended median survival rates [170]. The findings of a randomized trial of Racotumomab indicated tumor cell necrosis [171]. Racotumomab (Vaxira), the first anti-idiotypic vaccine, has received regulatory approval and is now available in both Cuba and Argentina. Vaxira has been shown to increase survival in patients with non-small cell lung cancer at recurrent or advanced stages (IIIB/IV). The Phase III study (NCT01460472) is currently in progress (Table 1).

Hence, numerous studies have demonstrated that tumor antigen-specific antibodies can facilitate antitumor activity by initiating an idiotypic cascade, which, in turn, stimulates an immune response specific to the tumor antigen.

### 6.2. Anti-Idiotypic Vaccines Designed to Target Tumor-Associated Protein Antigens

Patients with advanced melanoma were treated with a mouse monoclonal antibody, MK2-23, that mimics HMW-MAA (high molecular weight melanoma-associated antigen). An immune response to HMW-MAA, decreased metastases, and extended overall survival were observed [172]. In two clinical studies, patients diagnosed with stage IV malignant melanoma received administrations of murine anti-Id mAb MF11-30, a mimetic of HMW-MAA. One patient exhibited complete remission with the resolution of several abdominal lymph nodes, and three patients demonstrated minor responses [173].

The effectiveness of two anti-Id mAbs, mAb 3H1 (CeaVac) and 11D10 (TriAb), which are designed to mimic carcinoembryonic antigen (CEA) and human milk fat globule protein (HMGF), respectively, was assessed in lung cancer patients. A Phase II study was performed using anti-Id mAbs 3H1 and 11D10 in patients with completely resected stage II and stage IIIA non-small cell lung cancer (NCT000000006470, Table 1).

Several clinical trials have reported the use of anti-Id mAb ACA-125, which imitates the CA-125 tumor antigen, characteristically overexpressed in ovarian tumors. Preclinical studies and Phase I clinical trials have indicated that ACA-125 can stimulate humoral and cellular immune reactions to CA-125, with no toxicity observed [174] (Table 1).

Studies on the use of anti-Id Abs as cancer vaccines in colorectal cancer have focused on three major tumor antigens of protein nature: EpCAM, CD55, and CEA [175]. Anti-Id mAb 3H1 was assessed in a controlled, randomized Phase III clinical trial involving 630 patients with untreated metastatic colorectal cancer [176]. Antibody responses to CEA were detected in 70% of individuals who received 3H1.

The anti-cancer vaccine 105AD7 is a human anti-Id mAb that functions similarly to CD55. A Phase I clinical trial demonstrated that the cancer vaccine improved the median relapse-free survival in vaccinated patients compared to those who were not [177]. However, these findings were not corroborated by a randomized, double-blind Phase II survival study [178] (Table 1).

Hence, anti-Id Abs might provide a basis for the creation of cancer immunotherapy vaccines.

### 6.3. Anti-Idiotypic HIV-1 Vaccines

Attempts to create an HIV-1 vaccine have been unsuccessful, even after decades of research [70,179]. An alternative method for developing an HIV-1 vaccine involves using anti-Id Abs.

Mouse mAbs specific to the viral protein p24 were used to generate anti-Id Abs. When rats were immunized with anti-Id Abs, they developed considerable levels of antibodies that targeted the p24 protein [180]. Another study produced six monoclonal anti-Id Abs that targeted two mouse mAbs against p24: VIC5 and VIC6. Mice given the combined antibodies showed only a slight reaction to the p24 antigen [181].

One strategy for creating an HIV-1 vaccine involves generating antibodies that target conserved areas on Env, like the CD4 binding site on gp120 (CD4bs). Using the concept of idiotypic mimicry, anti-Id Abs were generated to mimic the conformation of CD4b. Rabbits that were vaccinated with these anti-Id Abs developed antibodies that could identify both recombinant and cellular human CD4 [182].

Another investigation yielded two murine anti-Id mAbs that mimicked the CD4 gp120 binding site. These anti-idiotype mAbs have demonstrated the capacity to recognize the idiotype of the broadly neutralizing human mAb b12 and elicit a robust response against gp120 in rabbits. Moreover, rabbit serum has been demonstrated to neutralize two susceptible HIV-1 strains in a pseudovirus neutralization assay [183].

Monoclonal antibody 2F5 is among the most potent and broadly neutralizing antibodies against HIV-1. Immunization with anti-Id mAb Ab2/3H6 has been proposed as an alternative method for the induction of 2F5-like antibodies. Specific antibodies to HIV-1 were produced in rabbits immunized with anti-Id mAba. However, these antibodies did not exhibit neutralizing activity against the virus [184].

An alternative strategy involved isolating broadly neutralizing IgG antibodies from HIV-infected individuals who maintained high CD4 and CD8 levels without specific therapy. The immunization of mice with these antibodies allowed two monoclonal antibodies against the idiotype, designated P1 and P2, to be isolated. The rabbit serum, produced following immunization with P1 antibody, exhibited neutralizing effects against HXB2 pseudovirus [183].

Another study tested four different formats of anti-Id mAb P1: two as single chains and two in the form of minibodies. A particular conformation of the mini-antibody MbVHVL has been found to have a high binding affinity for the broadly neutralizing antibody b12 and to stimulate an immune response to HIV-1 in rabbits [185].

Several studies of the immune response to HIV-1 have documented the presence of antibodies with the common idiotype designated as 1F7. Administration of monoclonal antibodies against 1F7 to macaques resulted in idiotype-specific clonal suppression with expansion of the antibody response to HIV envelope proteins [186].

Another study involved the creation of anti-Id mAb iv8, which has a strong binding affinity for VRC01 class B-cell precursor receptors. It has been demonstrated that anti-Id mAbs have the potential to increase VRC01 class B cells in vivo while avoiding the activation of B cell clones causing off-target responses to Env [187].

Hence, anti-idiotypic immunogens can be generated using the idiotype of broadly neutralizing antibodies. Anti-idiotypic antibodies can serve as a basis for creating novel HIV vaccine ideas.

### 6.4. Anti-Idiotypic Antibody-Based Vaccines: Prospects and Challenges

The progress in developing oncology vaccines based on anti-Id Abs is very encouraging. A number of vaccines are either approved for use or in different phases of clinical trials. For example, a Phase Ib clinical trial employing anti-Id Abs demonstrated efficacy in twenty-three patients with advanced colorectal cancer, who had not responded to standard treatments [188]. Clinical trials have shown that the anti-cancer drug racotumomomab is safe and well-tolerated, and it has been authorized for use in Cuba and Argentina under the name Vaxira [189]. In 2010, the FDA approved sipuleucel-T as the first cancer vaccine for treating metastatic hormone-refractory prostate cancer [190]. In 2015, the FDA and EMA approved and granted orphan drug status to dinutuximab (sold as unituxin) for use in treating high-risk neuroblastoma in children as a first-line treatment [191]. Dinutuximab β (marketed under the name isquette) gained approval in 2017 as a secondary treatment option for neuroblastoma [192].

With regard to autoimmune diseases, the use of anti-Id Abs as vaccines in patients is still in its infancy. Only a small number of drugs are entering the clinical trial phase. A small clinical trial, for example, demonstrated a positive outcome in SLE patients who received vaccination with anti-Id Abs [193].

Table 1 provides an overview of anti-Id Abs-based vaccines currently in different phases of clinical trials, with the information sourced from https://clinicaltrials.gov/ (accessed 14 August 2025).

However, the development of vaccines based on anti-Id Abs faces a number of challenges. The process of obtaining high-quality and standardized anti-Id Abs is multistage and costly. Antibodies can be produced in monoclonal or polyclonal forms, each with distinct limitations in their manufacturing processes. Monoclonal antibodies are produced through hybridoma technologies, which demand considerable technical proficiency and prolonged manufacturing periods [194]. For example, such technology was used to generate a murine anti-Id mAb that simulates a specific epitope of carcinoembryogenic antigen, a known tumor-associated antigen [195].

Polyclonal antibodies can be derived from immunized animals. Nevertheless, the unique biochemical and biophysical properties of these antibodies may exhibit inter-batch variability, which poses a challenge to their utilization as pharmaceuticals [189]. A polyclonal anti-idiotypic antibody developed in goats against CO17-1A/GA733 binds to two different epitopes of the colorectal cancer antigen, in contrast to monoclonal antibodies that bind to only one epitope of the antigen [196]. Despite batch-to-batch variability, polyclonal antibodies can be produced in a shorter timeframe compared to monoclonal antibodies.

Interest in idiotypic vaccines has waned somewhat with the introduction of mRNA and DNA vaccine platforms that offer faster and more economical development pathways.

Despite the considerable obstacles associated with developing anti-Id Abs as vaccines, ongoing advances in research and clinical trials are producing encouraging outcomes, indicating that the potential applications of anti-idiotypic vaccines might be substantially broadened. The principle of using anti-Id Abs represents a brilliant example of how the internal regulatory mechanisms of the immune system can be harnessed against itself to generate protection. This strategy enables the immune system to develop defenses against genuine threats by presenting it with nothing more than a precise mold of the target. This field continues to remain an important niche in immunology and biotechnology, particularly for addressing particularly challenging tasks.

## 7. Conclusions

The application of anti-Id Abs in treating several diseases presents a highly promising strategy. In the field of oncology, the therapeutic potential of anti-Id Abs can be harnessed to treat a variety of cancers. Tumor-associated antigens can be used to induce a strong immune response with negligible side effects instead of chemotherapy or radiation therapy. The primary benefit of the anti-idiotypic strategy is the specific targeting of tumor antigens, which results in reduced toxicity and fewer side effects compared with conventional treatments.

Gaining insight into the mechanisms governing immune response regulation in autoimmune diseases will yield considerable clinical advantages for diagnosis and treatment. The ability of anti-Id Abs to neutralize and inhibit the secretion of pathological autoantibodies may find application in the development of therapies. A major advantage of using anti-Id Abs therapies is their ability to precisely target complementary autoantibodies, thus successfully reducing the specific immune response without compromising the normal function of the rest of the immune system.

Anti-Id Abs can be used in the treatment of other diseases as well. We conclude that the current technological and equipment infrastructure permits the development of innovative methodologies for the treatment, diagnosis, and prevention of a range of diseases, based on anti-Id Abs. Furthermore, investigations into anti-Id Abs will enhance our comprehension of immune system regulation and offer new approaches to targeted therapy.

## Figures and Tables

**Table 1:** Anti-Id Abs, in different phases of clinical trials. The data were sourced from https://clinicaltrials.gov/ (accessed 14 August 2025).

Status	Antibody	Official Title	Conditions	Last Update Posted	ClinicalTrials.gov ID
Completed	BEC2	The SILVA Study: Survival in an International Phase III Prospective Randomized LD Small Cell Lung Cancer Vaccination Study with Adjuvant BEC2 and BCG	Lung Cancer	6 March 2012	NCT00003279
Unknown status	Racotumomab	A Prospective, Randomized, Multicenter, Open Label Phase III Study of Active Specific Immunotherapy with Racotumomab Plus Best Support Treatment Versus Best Support Treatment in Patients with Advanced Non-small Cell Lung Cancer.	NSCLC Lung Cancer, Non-small Cell	29 July 2016	NCT01460472
Completed	3H1	Phase II Study of Postoperative Adjuvant Immunotherapy and Radiation in Patients with Completely Resected Stage II and Stage IIIA Non-Small Cell Lung Cancer	Lung Cancer	13 August 2013	NCT00006470
Completed	ACA-125	A Phase I/II Trial of ACA 125 in Patients with Recurrent Epithelial Ovarian, Fallopian Tube, or Peritoneal Cancer	Ovarian Cancer Fallopian Tube Neoplasms Peritoneal Neoplasms	2 October 2006	NCT00103545
Completed	ACA-125	Phase I Trial of the Monoclonal Anti-Idiotype Antibody ACA125 in Patients with Epithelial Ovarian, Fallopian Tube, or Peritoneal Cancer	Fallopian Tube Cancer Ovarian Cancer Primary Peritoneal Cavity Cancer	5 June 2013	NCT00058435
Unknown status	105AD7	A Phase I/II Trial of an Allogeneic Cell-Based Vaccine and an Anti-Idiotypic Antibody Vaccine Approach for Metastatic Adenocarcinoma of the Colon or Rectum	Colorectal Cancer	20 September 2013	NCT00007826
Completed	4B5	A Phase I/II Trial of a Human Anti-Idiotypic Monoclonal Antibody Vaccine (4B5) Which Mimics the GD2 Antigen, in Patients with Melanoma	Melanoma (Skin)	12 April 2013	NCT00004184

## Data Availability

The original contributions presented in this study are included in the article. Further inquiries can be directed to the corresponding authors.

## Abbreviations

The following abbreviations are used in this manuscript:

Ab	Antibody
ADAs	Anti-drug antibodies
anti-Id Abs	Anti-idiotypic antibodies
CD4bs	CD4 binding site
CDR	Complementarity-determining region
dsDNA	Double-stranded DNA
ELISA	Enzyme-linked immunosorbent assay
EMA (EMA)	European Medicines Agency
FDA	Food and Drug Administration
GAD65	65-kDa isoform of glutamate decarboxylase
HMGF	Human milk fat globule protein
LC-MS/MS	Liquid chromatography and tandem mass spectrometry
mAb	Monoclonal antibody
NOD	Non-obese diabetic
RBD	Receptor-binding domain
S	Spike
SLE	Systemic lupus erythematosus
VH	Variable domain
